# Mechanistic insight into the mode of inhibition of dietary flavonoids; targeting macrophage migration inhibitory factor

**DOI:** 10.3389/fmolb.2024.1414572

**Published:** 2024-06-10

**Authors:** Ali Raza Siddiqui, Mamona Mushtaq, Madiha Sardar, Lubna Atta, Mohammad Nur-e-Alam, Aftab Ahmad, Zaheer Ul-Haq

**Affiliations:** ^1^ H. E. J. Research Institute of Chemistry, International Center for Chemical and Biological Sciences, University of Karachi, Karachi, Pakistan; ^2^ Dr. Panjwani Center for Molecular Medicine and Drug Research, International Center for Chemical and Biological Sciences, University of Karachi, Karachi, Pakistan; ^3^ Department of Pharmacognosy, College of Pharmacy, King Saud University, Riyadh, Saudi Arabia; ^4^ Department of Biomedical and Pharmaceutical Sciences, Chapman University School of Pharmacy, Irvine, CA, United States

**Keywords:** macrophage migration inhibitory factor, dietary flavonoids, molecular dynamic simulation, principal component analysis, ISO-1

## Abstract

**Introduction:** The Macrophage Migration Inhibitory Factor (MIF), a key pro-inflammatory mediator, is responsible for modulating immune responses. An array of inflammatory and autoimmune diseases has been linked to the dysregulated activity of MIF. The significance in physiological as well as pathophysiological phenomena underscores the potential of MIF as an attractive target with pharmacological relevance. Extensive research in past has uncovered a number of inhibitors, while the ISO-1, or (S, R)-3-(4-hydroxyphenyl)-4,5-dihydro-5-isoxazole acetic acid methyl ester being recognized as a benchmark standard so far. Recent work by Yang and coworkers identified five promising dietary flavonoids, with superior activity compared to the standard ISO-1. Nevertheless, the exact atomic-level inhibitory mechanism is still elusive.

**Methods:** To improve the dynamic research, and rigorously characterize, and compare molecular signatures of MIF complexes with ISO-1 and flavonoids, principal component analysis (PCA) was linked with molecular dynamics (MD) simulations and binding free energy calculations.

**Results:** The results suggest that by blocking the tautomerase site these small molecule inhibitors could modify the MIF activity by disrupting the intrinsic dynamics in particular functional areas. The stability matrices revealed the average deviation values ranging from 0.27–0.32 nm while the residue level fluctuations indicated that binding of the selected flavonoids confer enhanced stability relative to the ISO-1. Furthermore, the gyration values extracted from the simulated trajectories were found in the range of 1.80–1.83 nm.

**Discussion:** Although all the tested flavonoids demonstrated remarkable results, the one obtained for the potent inhibitors, particularly Morin and Amentoflavone exhibited a good correlation with biological activity. The PCA results featured relatively less variance and constricted conformational landscape than others. The stable ensembles and reduced variation in turns might be the possible reasons for their outstanding performance documented previously. The results from the present exploration provide a comprehensive understanding of the molecular complexes formed by flavonoids and MIF, shedding light on their potential roles and impacts. Future studies on MIF inhibitors may benefit from the knowledge gathered from this investigation.

## 1 Introduction

Discovered almost 4 decades ago while studying the Type IV hypersensitivity reaction, macrophage migration inhibitory factor (MIF) stood out among the first recognized cytokines. MIF serves various roles and acts as a signaling molecule, that is, a cytokine, chemokine, hormone, molecular chaperone, or enzyme (Zhang et al., 2022). Being an essential effector molecule, MIF initiates and coordinates the humoral and cellular responses, playing a central role in immunomodulation. MIF is regarded as a potential biomarker for several diseases presenting with inflammatory elements because it regulates both adaptive and innate responses. The multifaced roles of the MIF and its involvement in the genesis of pathologies like sepsis, cancer, and autoimmune diseases are highlighted in the literature, rendering MIF an attractive target for drug development (Kang and Bucala, 2019). The biological effects elicited by MIF are mediated by both intracellular and extracellular mechanisms. While the intracellular pathway, which is receptor independent, works by physical interaction with an array of resident proteins and enzymes, the extracellular signaling involves binding of MIF to receptor (CD74)/co-receptor complexes (CD44) (Neo and Mitchell, 2020). The attachment of MIF to the receptor activates multiple signaling cascades mediated by two mechanisms involving MAP kinase (p38, JNK, and ERK1/2) and phosphatidil-inositol-3 kinase (PI3K) dependent pathways. The process eventually results in the migration of various transcription factors, like AKT, NF-κB, and STAT3, inside the nucleus, thereby instigating the activation of genes associated with inflammation (Vázquez et al., 2023).

MIF is highly conserved among vertebrates (but is also found in arthropods, nematodes, and protozoans). MIF weighs 12.5 kDa, comprises 114 residues, and has a toroidal shape (Bilsborrow et al., 2019; Penticuff et al., 2019). In its functional form, MIF manifests as a homotrimeric configuration. Each monomer consists of two antiparallel α-helices positioned closely alongside a four-stranded β sheet. The assembly gains additional stability via the interaction of β-strands and sheets of nearby subunits forming the interface region. In general, the structure features a central channel accessible to solvent and a barrel-shaped motif, while the catalytic region is located at the interface of two adjacent monomers (Zhang et al., 2022; Zhang et al., 2024). In addition to involvement in immune responses, MIF functions as thiol-protein oxidoreductase, which depends on a four-residue motif, Cys–Ala–Leu–Cys(CXXC), occupying 56–59th position. The Cys-59 is essential for the underlying activity (Trivedi-Parmar et al., 2018; Qian et al., 2019; Mahalingam et al., 2020). Moreover, the protein under discussion also executes the phenylpyruvate tautomerase activity, catalyzing the tautomerization of phenylpyruvate tautomerase and D-dopachrome, dependent on the distinctive proline residue (Pro1) from the N-terminal of the monomer. Thus, Pro1 is critical for the interaction of MIF with other receptors on the cell surface (Yang et al., 2021; Chen et al., 2023). However, uncertainties exist concerning the correlation between enzymatic activity and physiological role. Thus, inhibitors that modify the surface characteristics of MIF remain a promising area for further exploration. In this regard, targeted efforts to inhibit the tautomerase activity of the enzyme by means of small molecules are a promising strategy, supported by experimental findings from both laboratory-based assays and animal models (Trivedi-Parmar and Jorgensen, 2018; O’Reilly et al., 2016; Sinitski et al., 2019).

**FIGURE 1:**
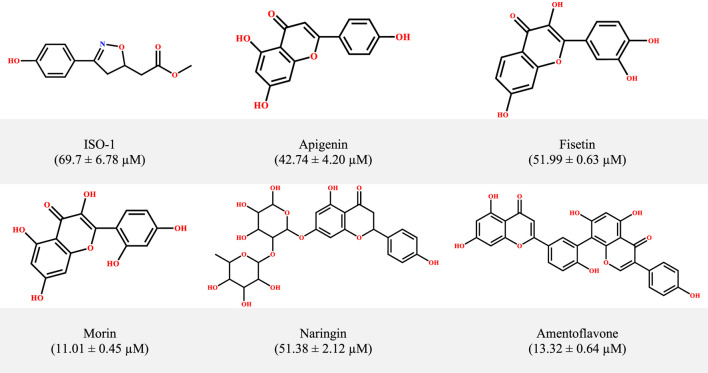
The structural information of MIF inhibitors, a subset within the small-molecule class of flavonoids, with corresponding inhibition concentrations (IC_50_) provided in parentheses.

From the initial identification of the reference inhibitor ISO-1, several small molecules have been suggested as potential inhibitors of MIF from different scaffolds, that is, pyrazole derivatives, biaryltriazoles, and benzoxazolones, working either as non-covalent or allosteric inhibitors (Lubetsky et al., 2002; Cournia et al., 2009; Jorgensen et al., 2010; Xu et al., 2014; Trivedi-Parmar et al., 2018). A growing body of studies has documented the potential of natural products such as curcumin, ellagic acid, epicatechins, and flavonoids (Dickerhof et al., 2014; Young et al., 2014; Sarkar et al., 2015; Sun et al., 2022). Previous studies, including the work of Garai and coworkers as an example, revealed the efficiency of the flavonoids luteolin and quercetin in interfering with the tautomerase activity of MIF (Garai and Adlercreutz, 2004). Inspired by the anticancer and anti-inflammatory features of flavonoids (Dai et al., 2012; Tuli et al., 2023), Yang and coworkers investigated a set of 24 flavonoids to assess their potential in counteracting both the tautomerase and the biological effects of MIF in the RAW264.7 cell line. They identified five promising candidates (Figure 1) with superior activity and low inhibition constants (IC_50_) relative to ISO-1. However, the precise mechanism for the observed inhibition for these flavonoids, particularly for the standout candidates, that is, morin and amentoflavone (IC_50_ = 11.01 ± 0.45 μM and 13.32 ± 0.64 μM, respectively), remains to be determined. The evolution and emergence of advanced *in silico* approaches have enabled the streamlining of the drug discovery pipeline alongside unraveling mechanistic insights. The precision and efficiency of *in silico* tools such as molecular dynamic simulation provide a wealth of benefits in understanding the actions of molecules at the atomic scale (Yuan et al., 2022). Much literature highlights the potential of computational explorations, particularly MD simulations, in revealing the mechanism of inhibition. This is exemplified by the previous work of Qureshi et al. (2022), along with many others. In a recent work, Baidya et al. (2023) used computational tools to provide a mechanistic insight into the inhibitory mechanism of proton pump inhibitors targeting choline acetyltransferase. A computational framework has been devised in the present work to uncover both the binding modes and time-dependent stability patterns for the mentioned dietary flavonoids. Following the exploration of binding modes in static and dynamic phases, the ligand-induced structural alterations triggered within the MIF were monitored at local and global levels. The second phase entails the computation of associated variance through PCA aimed at exploring the conformational landscape.

The finding from the current work provides a comprehensive molecular-level insight into the MIF-flavonoids and ISO-1 complexes, thus shedding light on the possible reasons for the observed differences in functionalities among the tested candidates. These insights are proposed to provide valuable information that might assist in the development of MIF inhibitors.

## 2 Methodology

The present work aims to identify the binding modes of the experimentally validated dietary flavonoids: apigenin (Ap1), fisetin (F9), morin (M12), naringin (N16), and amentoflavone (Am22), in the tautomerase site of MIF. It will be followed by the structural characterization, thereby elucidating the mechanism of inhibition via a detailed biophysical analysis.

### 2.1 Structure preparation

The atomic coordinates of MIF in complex with ISO-1, corresponding to the PDB ID: 1LJT (r: 0.237), were retrieved from the repository of the Protein Data Bank (Lubetsky et al., 2002). The unwanted heteroatoms and the chains other than A and B were deleted. The initial preparatory steps involved the correction and protonation at the default settings in Molecular Operating Environment (MOE) version 2019 (MOE, 2019) (Chemical Computing Group Inc., 2019). Subsequently, the protein was subjected to energy minimization and assignment of partial charges using the Amber10EHT force field. The selected ligands were constructed in ChemDraw version 22.0.0, followed by preparation using MOE. In brief, lone pairs were adjusted, and hydrogens were added where required via the “Protonate 3D” module. The minimization in terms of energy and the assignment of partial charges were in accordance with the MMFF94X force field. The dielectric constant was set to 1, while the RMS gradient threshold was set to 0.1 kcal/mol/Å. The optimized compounds were then subjected to docking studies.

### 2.2 Molecular docking studies of inhibitors against MIF

The active or tautomeric site is reported to be positioned at the interface of two monomers. Initially, the the docking software and protocol were benchmarked via a redocking strategy. The resultant deviation in turns indicate the fitness of the docking platform, determining the appropriate use for further work. Based on the benchmarking, the docking was executed with MOE. The “Alpha Triangle” placement method rendered the placement of the ligand poses in the targeted cavity. The resultant poses were scored with the GBVI/WSA dG scoring function (SF), following refinement with the London dG SF. The induced fit docking approach was used, thereby allowing some mobility (Khan et al., 2021; Sardar et al., 2022). Of the retained poses, the conformation featuring the lowest score was selected for interaction pattern analysis via the Protein-Ligand Interaction Profiler (PLIP) (Salentin et al., 2015). The intermolecular interaction patterns alongside the binding modes were modeled and pictured using UCSF Chimera (Pettersen et al., 2004).

### 2.3 Molecular dynamic simulations

In order to examine the kinetic and structural interactions present in the protein–ligand complexes, a 100 ns molecular dynamics (MD) simulation for six different systems was performed using the Gromacs 2021 software package (Abraham et al., 2015). The ACPYPE (Ante-Chamber PYthon Parser interface) server was used to create the ligand topologies described by Sousa da Silva and Vranken (2012). Meanwhile, the Amber ff99SB-ILDN force field was utilized for the target protein parameterization. The ligands and receptors were then combined to form a single, cohesive system, and each resulting complex was enclosed in a box with a cubic shape. Careful positioning was done to ensure that any solute atoms were at least 10Å away from the margins of the periodic box. The TIP3P (transferable intermolecular potential 3P) water model was used to solve the systems (Mark and Nilsson, 2001). Counter ions (Na^+^ and Cl^−^) were added to maintain system neutrality. The initial phase consisted of a 50,000-step energy reduction procedure designed to remove steric collisions. The particle-mesh Ewald (PME) approach was utilized to handle long-range electrostatic interactions, whereas van der Waals forces and short-range electrostatic interactions were terminated at 1.0 nm. Following successful convergence, all systems were subjected to pressure equilibration at 1 bar via the NPT ensemble (isothermal and isobaric) and temperature equilibration at 300 K under the NVT condition (constant temperature and volume), using periodic boundary conditions, a Parrinello–Rahman barostat, and a Berendsen thermostat. Following system equilibration, a 100 ns production run was applied. The real-time dynamics and stability of every complicated system were observed using the leap-frog integrator, which applied a time step of 2 fs. The radius of gyration (RoG), root mean square deviation (RMSD), and root mean square fluctuation (RMSF) were the stability indices that were used to track the stability pattern, followed by principal component analysis (PCA). Visual molecular dynamics (VMD) (Humphrey et al., 1996) was used for visual assessment, and the Xmgrace tool (Turner, 2005) was used to plot the graphs.

### 2.4 Collective motion; principal component analysis

PCA is a potent statistical technique for extracting important information from large datasets. In this investigation, we want to use PCA to find and examine possible differences in a complex’s energetic and structural characteristics. The focus is on using the MDAnalysis tool (Michaud-Agrawal et al., 2011; Gowers et al., 2016) to analyze simulated data. This study focuses on five flavonoids and the ISO-1 standard, exploring their principal components. The goal is to identify subtle variations in these molecules’ dynamic behavior that may emerge during the MD simulations. Using PCA, we aimed to isolate and emphasize the key elements influencing the observed alterations in the complex’s properties. This strategy provides a means of identifying patterns and trends in the complex interactions between molecule energy and structures.

### 2.5 Binding free energy calculation

When combined with techniques for calculating free energy, molecular dynamics can yield informative energetic determinants of binding interactions. The binding free energy, or ΔG bind, is used to quantify the strength of the binding. As a result, the combination of molecular mechanics and generalized Born surface or Poisson–Boltzmann (MMGB/PBSA) has become a popular, dependable, and effective method for doing free energy simulations, especially when dealing with protein–ligand binding (Genheden and Ryde, 2015). Molecular mechanics/generalized Born surface area (MM/GBSA) was adopted in this work to evaluate the binding free energies of the selected flavonoids and the ISO-1 standard. For every simulated system, 1,000 frames were taken from the final 10 ns of the molecular dynamics trajectory. Furthermore, separate computations of different energy components were performed, such as electrostatic energy, van der Waals interactions, and polar and nonpolar solvation energies. Together, these elements aid in the thorough calculation of the total binding free energy. The calculations performed were as follows.

$$∆G_{bind}=G_{complex}-\;G_{protein}-\;G_{ligand}$$

$$∆G_{bind}=∆E_{MM}+∆G_{GB}+∆G_{SA}-T∆S$$

$$∆G_{bind}=∆E_{vdw}+∆E_{ele}+∆G_{GB}+∆G_{SA}-T∆S$$

where $∆E_{MM}$ is the gas-phase interaction energy between protein–ligand complex, the van der Waals energy contribution is $∆E_{vdw}$ , the electrostatic energy contribution is $∆E_{ele}$ , and the free energy for polar and nonpolar components of desolvation is $∆G_{GB}\;and∆G_{SA}$ . ∆S is the entropy contribution at a given temperature (T). Binding energy calculations were calculated using the gmx_MMPBSA tool (Valdés-Tresanco et al., 2021).

## 3 Results and discussion

Serving an array of biological functions, MIF acts as a signaling molecule and influences both arms of the immune responses. The pleiotropic nature thus renders MIF a prospective biomarker and a promising pharmacological target for several etiologies demonstrating inflammatory elements (Kang and Bucala, 2019; Zhang et al., 2022). Yang et al. (2021) evaluated a series of 24 flavonoids to assess their inhibitory potential toward MIF. Five flavonoids with low IC_50_ values and, hence, superior potency to the known standard (ISO-1) were identified. However, the precise reason for the observed inhibition pattern remains elusive. The development of advanced computational tools (especially advanced molecular docking and MD simulation methods) allows for the accurate investigation of complex inhibitory mechanisms, transforming the field of drug discovery and enhancing our comprehension of biomolecular interactions (Rossetti and Mandelli, 2022; Jin and Wei, 2024). In an attempt to provide a detailed insight, a computational exploration has been made to envisage the binding conformations both in static and dynamic modes. The comprehensive findings outline the time-dependent conformational alterations, stability patterns, and dynamic interactions triggered by the binding of the ligand with the protein of interest. These understandings can be an important guide for future efforts to build MIF inhibitors.

### 3.1 Molecular docking simulation: an insight into binding modes

**FIGURE 2:**
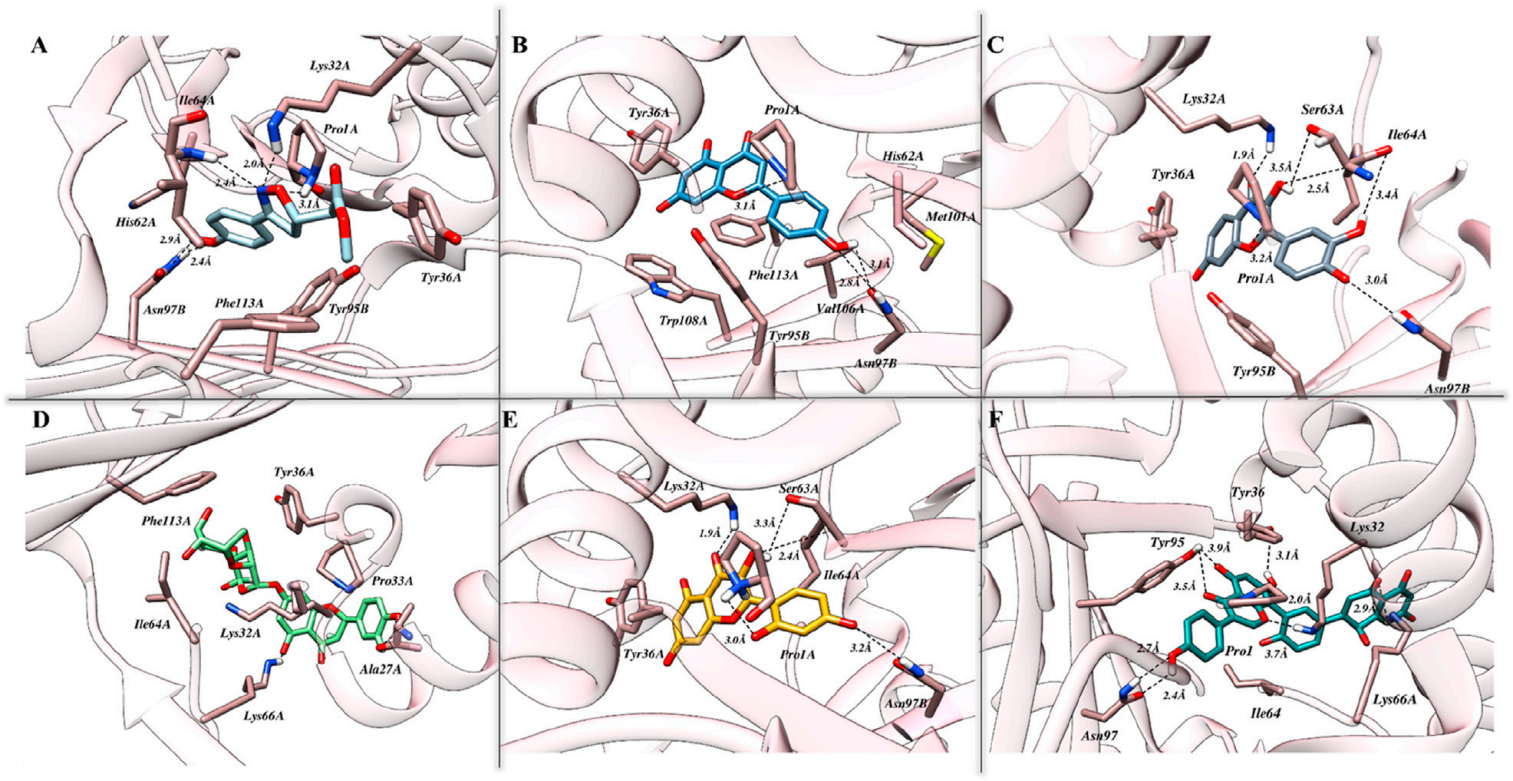
The intermolecular interaction patterns of MIF in complex with **(A)** ISO-1, **(B)** Ap1, **(C)** F9, **(D)** N16, **(E)** M12, and **(F)** Am22 flavonoids are shown in a three-dimensional depiction. Dotted black lines are used to depict hydrogen bonds, while sticks are used to symbolize the involved residues.

The exploration of binding modes driven by molecular docking studies was initiated following the benchmarking of both the protocol and the docking station. As presented in Supplementary Figure S1, promising efficiency was demonstrated by London dG, GBVI/WSA dG, and Alpha Triangle as a preferred method for the initial placement of the resultant conformations and primary and secondary scoring functions, respectively. Identified originally, ISO-1 is known for its high specificity and hence used as the reference inhibitor for MIF. The binding cavity native to ISO-1 lies close to the CD74 receptor binding site and is surrounded by Y36, K66, and N109. The docking results, in correlation with biological activity, highlighted strong affinities of tested flavonoids toward MIF, with Am22 and F9 standing out among others. Docking scores of −15.28 kcal/mol and −15.39 kcal/mol were yielded for M12 and Am22, featuring the lowest IC50 values of 11.01 ± 0.45 μM and 13.32 ± 0.64 μM. The N16, Ap1, and F9, on the other hand, were presented with docking scores of −12.26 kcal/mol, −13.22 kcal/mol, and −15.91 kcal/mol, respectively, compared to the −11.95 kcal/mol recorded for ISO-1. The interaction patterns observed for the protein–ligand complexes are presented in Figure 2. Supplementary Table S1 suggested the contribution of both the hydrophobic and electrostatic contacts in providing overall stability to the complexes.

### 3.2 Exploring mechanistic insight: a comparative study of ISO-1 and dietary flavonoids bound MIF

MD simulations represent a conventional approach to producing physically relevant ensembles of conformations. The generation of conformational ensembles relies on employing complementary force fields, describing the forces acting on atomic components. Herein, an all-atom dynamic simulation was conducted involving the ligand-bound complexes of MIF. In the following portions, an in-depth analysis of the stability patterns and the associated variations in the resulting conformations of the simulated systems is presented. The following sections will detail the perturbations in stability patterns and conformational landscape following the binding of the dietary flavonoids.

#### 3.2.1 An insight into the trend of global variations

**FIGURE 3:**
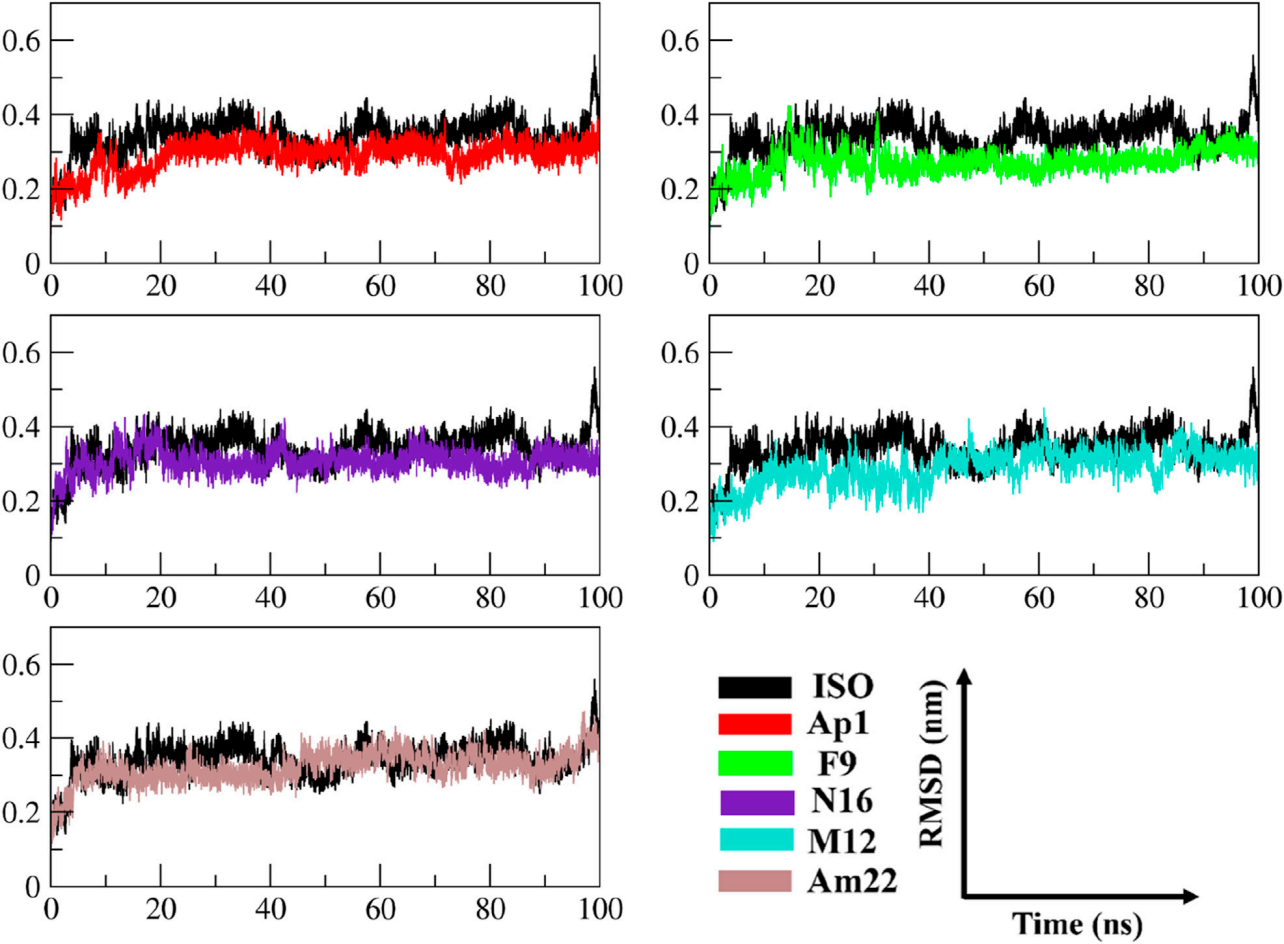
The time evolution of the RMSD between MIF’s bound states with dietary flavonoids and ISO-1 shed light on the protein’s structural stability and conformational changes.

The multitude of conformations resulting from the simulation studies necessitates the clustering of conformations using a common metric in order to monitor the dynamical changes experienced by the simulated system with respect to time (Sargsyan et al., 2017). The RMSD, a pairwise similarity metric, was employed to compute the differences in backbone coordinates. As illustrated in Figure 3, the computation revealed that a well-converged and equilibrated state was observed following an initial phase of stabilization. The initial ∼40 ns presented substantial backbone deviations ranging from 0.25 nm to 0.32 nm, reaching a maximum height of 0.43 nm observed in the case of ISO-1-bound MIF. Secondary to this was the N16-bound system, which presented a maximum deviation peak of 0.43 nm. The ISO-1 induced significant deviations both during the initial and following the attainment of the stabilization compared to the selected flavonoids. However, an overlapping trend was noted during the last 10–20 ns among the systems except for the ISO-1- and Am22-bound MIF, which featured altered patterns (Supplementary Figure S2). Average pairwise backbone deviations of 0.28 ± 0.02 nm, 0.30 ± 0.02 nm, 0.31 ± 0.03 nm, 0.32 ± 0.03 nm, 0.34 ± 0.03 nm, and 0.35 ± 0.04 nm were computed for the converged ensemble. The calculation from the entirety of the trajectories resulted in the values of 0.27 ± 0.04 nm, 0.29 ± 0.04 nm, 0.31 ± 0.03 nm, 0.29 ± 0.05 nm, 0.32 ± 0.04 nm, and 0.34 ± 0.05 nm for F9, AP1, N16, M12, Am22, and ISO-1, respectively. The increased deviations recorded for the flavonoids N16 and Am22 are likely due to the structural complexity and associated size, hindering the proper fitting within the binding cavity. The distribution patterns for the RMSDs highlighted enhanced stability to the receptor compared to the benchmark standard.

#### 3.2.2 An in-depth exploration revealing the local dynamics

**FIGURE 4:**
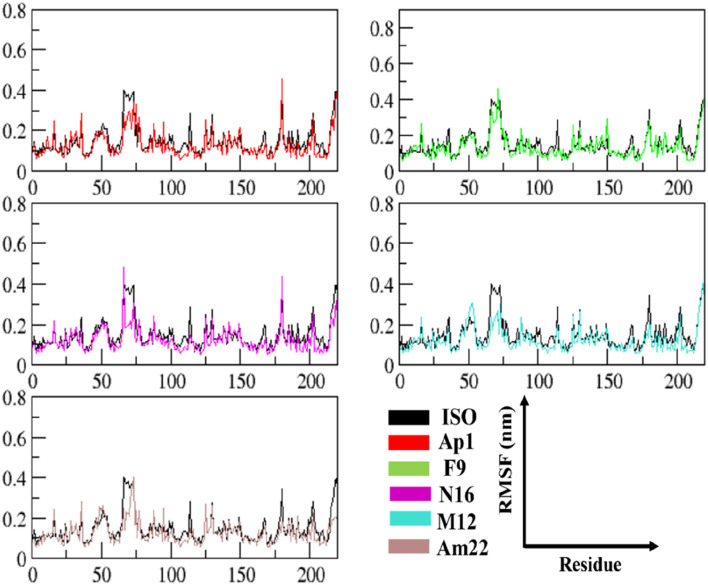
The analysis of time-dependent RMSF demonstrates conformational alterations and highlights MIF’s structural stability.

The RMSF data provide significant insights into the dynamics of constituent amino acids harbored by the macromolecular system. The perturbations triggered by the binding event consequently shed light on the binding behavior of the ligand (Zhang et al., 2020). The thorough evaluation of the residue-wise fluctuations via calculating the RMSF undergone by the backbone atoms of the protein unveiled a uniform degree of stability post-ligand binding. The outcome thus underscores the capability of these ligands to impart enhanced stability by restricting the mobility of the residues in most of the cases relative to the standard, as depicted in Figure 4. An overview of the fluctuation values recorded is presented in a box plot format to provide an insight into the range of the fluctuations experienced by the different systems of MIF (Supplementary Figure S3). The maximum and the minimum height of the fluctuations were observed at 0.837 nm and 0.055 nm and originated from the MIF when complexed with Ap1 and F9, respectively. Upon computation, the mean fluctuations were 0.14 ± 0.10 nm, 0.13 ± 0.08 nm, 0.13 ± 0.09 nm, 0.13 ± 0.06 nm, and 0.15 ± 0.12 nm for Ap1, N16, M12, Am22, and F9 compared to the value of 0.16 ± 0.10 nm for the ISO-1-MIF complex. Next, the focus was directed toward the particular residues lining the binding cavity. The binding pocket residues and fluctuations from both chains are presented in Supplementary Figure S4. As expected, the residues from MIF, when bound to dietary flavonoids, featured less fluctuation than ISO-1, highlighting their contribution in rendering the system stable. Limiting the protein’s mobility, hence, could be a likely mechanism for the underlying antagonistic effect of these flavonoids. This structural alteration, in consequence, impacts the normal function of the protein.

#### 3.2.3 Exploring the folding behavior and molecular compactness

**FIGURE 5:**
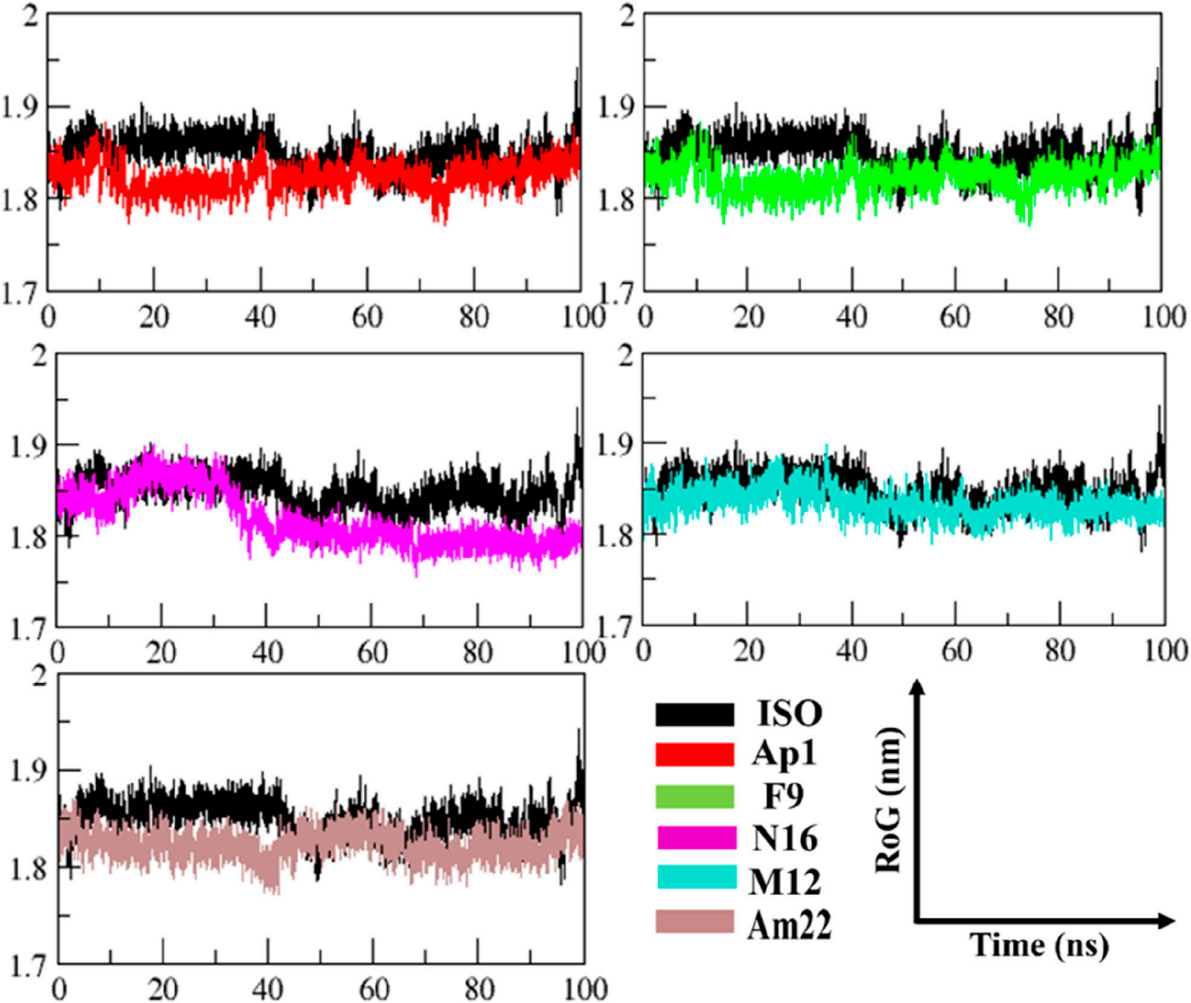
The pattern of gyration is shown in the MIF-simulated complexes.

The radius of gyration (RoG) was used to monitor stability in ligand-bound complexes of MIF, shedding light on the time-dependent folding behavior in the presence of solvent. The stabilization of gyration curves indicates that the protein is correctly folded, while fluctuations are associated with structural changes (Li D. D. et al., 2020). Thus, lower RoG values represent a compact and well-folded conformation, while the higher values are linked to loss of compaction and an unfolded state. As presented in Figure 5, the RoG curves level off after 44 ns of the run in general, with notable fluctuations in gyration values for the ISO-MIF complex. A closer view into the RoG trend during the converged phase (Supplementary Figure S5) featured an overlapping pattern for N16 and M12-MIF complexes with recorded mean values of 1.83 ± 0.01 nm. Similar to this were the observations made for Ap1 and Am22, both with an average value of 1.82 ± 0.01 nm. In line with the pattern observed for RMSD plots, notable fluctuations in the curve were seen for the N16-bound MIF compared to the rest of the systems under investigation. In contrast, the protein bound to the F9 attained a more folded state among all, followed by Am22. The mean values extracted from the curves, when considering the whole trajectories, were 1.80 ± 0.01 nm, 1.82 ± 0.01 nm, 1.83 ± 0.01 nm, 1.83 ± 0.01 nm, and 1.83 ± 0.01 nm for F9, Am22, AP1, N16, and M12, respectively.

Conversely, the value of 1.84 ± 0.02 nm was extracted for MIF bound to ISO-1. Note that all the test systems showcased a well-folded state, highlighting the structural integrity relative to the standard ligand used herein and signifying optimum convergence.

### 3.3 Intermolecular interaction pattern post-simulation

The binding of ligands to the cognate pocket is shaped by the intricate network of intermolecular interactions during the dynamics governing the conformational behavior and the stability of the complexes. Knowledge about these drivers provides a comprehensive insight into the structural characterization, functional relationship, and subsequent applications as therapeutics. In the succeeding section, the intermolecular interaction profile of the ligands inside the designated pocket is covered. The translocation in the initial placement observed for the hits following the simulation run can clearly be seen in Supplementary Figure S6, highlighting the ligand’s efficiency towards optimal accommodation in the binding cavity.

#### 3.3.1 Electrostatic interactions: a spotlight on hydrogen bond dynamics

**FIGURE 6:**
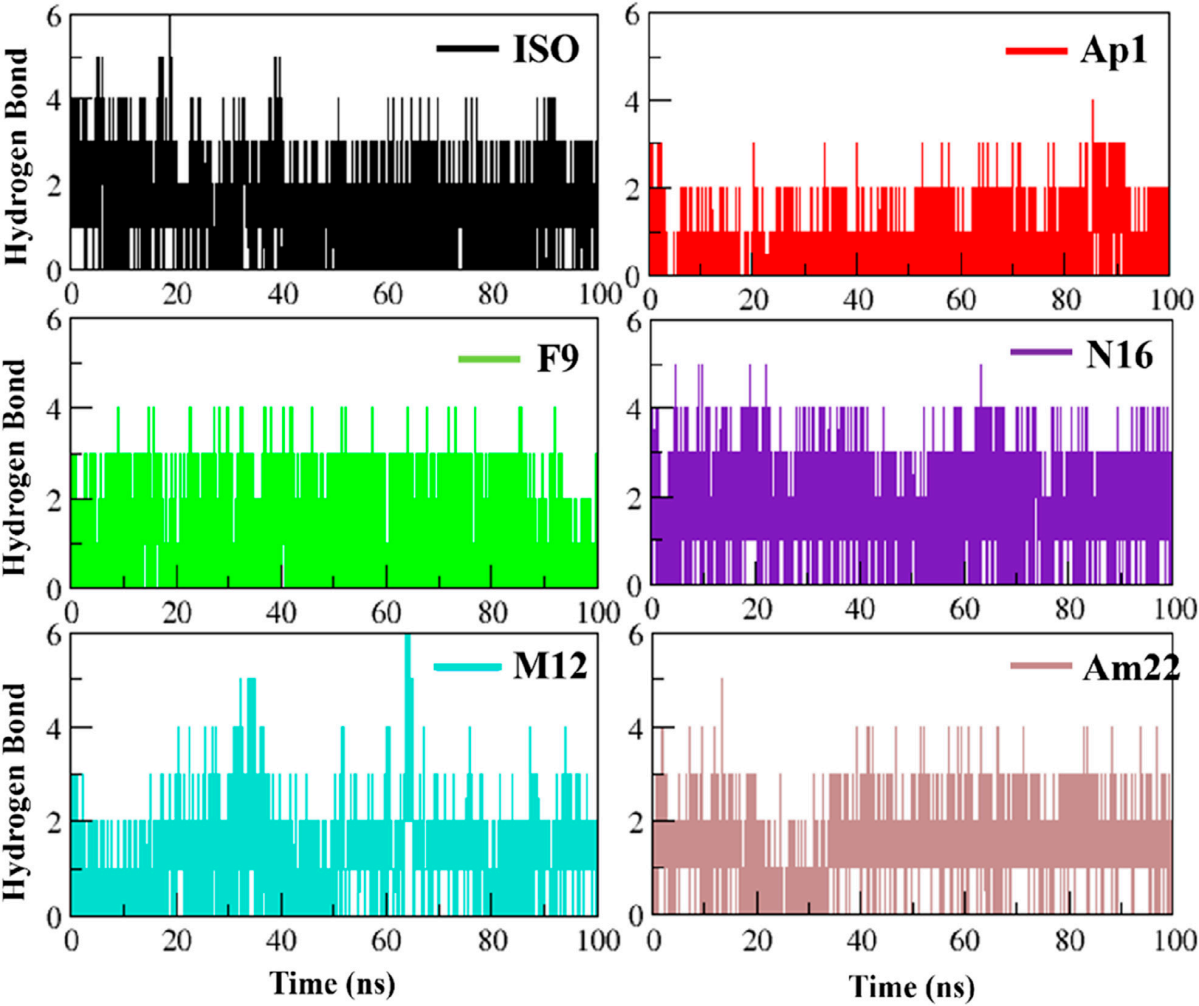
Plotting the evolution of hydrogen bonding over time for the selected dietary flavonoids.

Electrostatic interactions, especially hydrogen bonds, are significant regarding structure maintenance and overall stability. Further, alterations in the H-bond network have been linked to pathological conditions such as Parkinson’s (Li X. et al., 2020). In this context, the formation of intermolecular H-bonds and the associated occupancies were monitored over time for the studied systems (Figure 6). The system with standard inhibitor ISO-1 presented with the formation of six hydrogen bonds; however, only three were observed with significant occupancy. N16 and Am22 formed five bonds, and three and two of these bonds had good occupancy, respectively. The MIF bound to Ap1 formed four H-bonds, with two being consistent over time. The F9, on the other hand, formed four H-bonds, of which three persisted in the majority of the frames. In contrast, M12 formed six H-bonds, though only two remained consistent over time.

**FIGURE 7:**
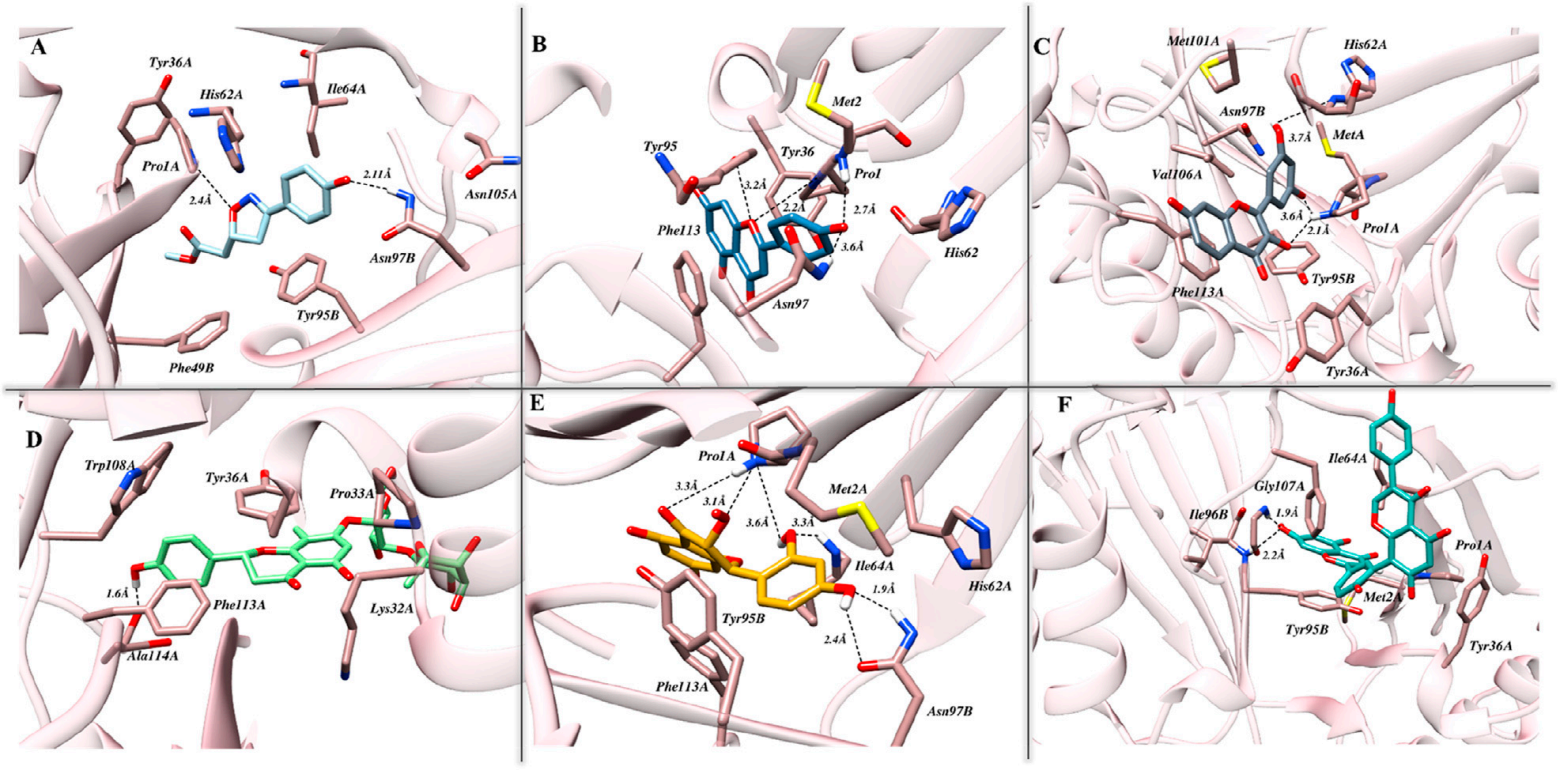
The pattern of intermolecular interactions between MIF in complex with **(A)** ISO-1, **(B)** Ap1, **(C)** F9, **(D)** N16, **(E)** M12, and **(F)** Am22 after the simulation run. Stick representations of the relevant residues are used, and dotted black lines show hydrogen bonding.

The interaction pose of ISO-1 and MIF, generated from the terminal frame revealed the formation of two electrostatic contacts, with protein residues acting as H-bond donors. Side chain amines of Pro1A and Asn97B donated their hydrogen atoms to the oxazole’s oxygen and the hydroxyl’s phenol oxygen and created H-bonds with lengths of 2.40 Å and 2.11 Å, respectively (Figure 7A). The test compound AP1 displayed five H-bonds where oxygen from the chromone moiety accepted a hydrogen from the pyrrolidine’s nitrogen of the Pro1A positioned 2.2 Å from the acceptor. Another contact of length 2.7 Å was established among the phenol’s oxygen, forming an H-bond of 2.7 Å with the amine nitrogen of Met2A. The carbonyl moiety of His62A, on the other hand, acted as an H-bond acceptor from the phenol, leading to the formation of a bond of length 3.5 Å. Further, TRP95 and Asn97B donated hydrogens to the resorcinol and pyrone ring of the ligand at 3.6 Å and 3.2 Å, respectively (Figure 7B). The stability of the F9 in the binding cavity was governed by three H-bonds. The Pro1A formed two H-bonds, acting as a donor and an acceptor for the reactive ligand moieties positioned 3.6 Å and 2.1 Å from the residue. Another H-bond of 3.7 Å was formed between the Ser63A’s amine and the resorcinol’s hydroxyl group (Figure 7C). The MIF bound to N16 retained only a single H-bond after the simulation run, where the carboxylic acid from the Ala114A’s backbone mediated interaction with the phenol 1.6 Å distant from the acceptor atom (Figure 7D).

The M12 featured the formation of six H-bonds, two involving amide groups (-CONH_2_) of Asn97B with bond lengths of 1.9 Å and 2.4 Å. The Pro1 from chain A interacted with the chromone moiety and established two H-bonds with lengths of 3.3 Å and 3.1 Å, and also with the resorcinol group featuring contact with a bond length of 2.2 Å. Additional stability was provided by the 3.3 Å contact between Ile64 from chain A and the ligand’s resorcinol (Figure 7E). Am22, on the other hand, acted as an H-bond acceptor for the Gly107A and Ile64B and mediated the formation of electrostatic contacts featuring bond distances of 1.9 Å and 2.2 Å, respectively (Figure 7F). It is important to highlight here that many electrostatic contacts were observed in docking studies, though not all remained persistent in the dynamic state. The alignment of binding poses before and after the simulation indicates that almost all the tested ligands experience translocation from the initial position. Because ligands are allowed to move freely in the presence of a solvent, the observed shift is not a strange phenomenon. This obvious shift in coordinates is proposed as a possible reason for the loss of the interaction observed under static mode. However, the ligands with a promising affinity towards the cognate protein do not leave the binding pocket, and the observed fluctuations underscore the mobility of the ligands during the stabilization phase when they try to fit in an optimal manner. ISO-1 and N16 experienced significant dislocation from the original coordinates, which justifies the loss of the initial contacts.

#### 3.3.2 Non-electrostatic contacts: an insight into the hydrophobic interactions

Overall, the interaction pattern observed for the dietary flavonoids highlighted their potential to efficiently engage the binding pocket residues via numerous hydrophobic contacts. Furthermore, some ligands also demonstrated π-π stacking, providing additional strength to the complexes. The details of the interactions, including the residues involved, are presented in Supplementary Table S2.

### 3.4 Principal component analysis

**FIGURE 8:**
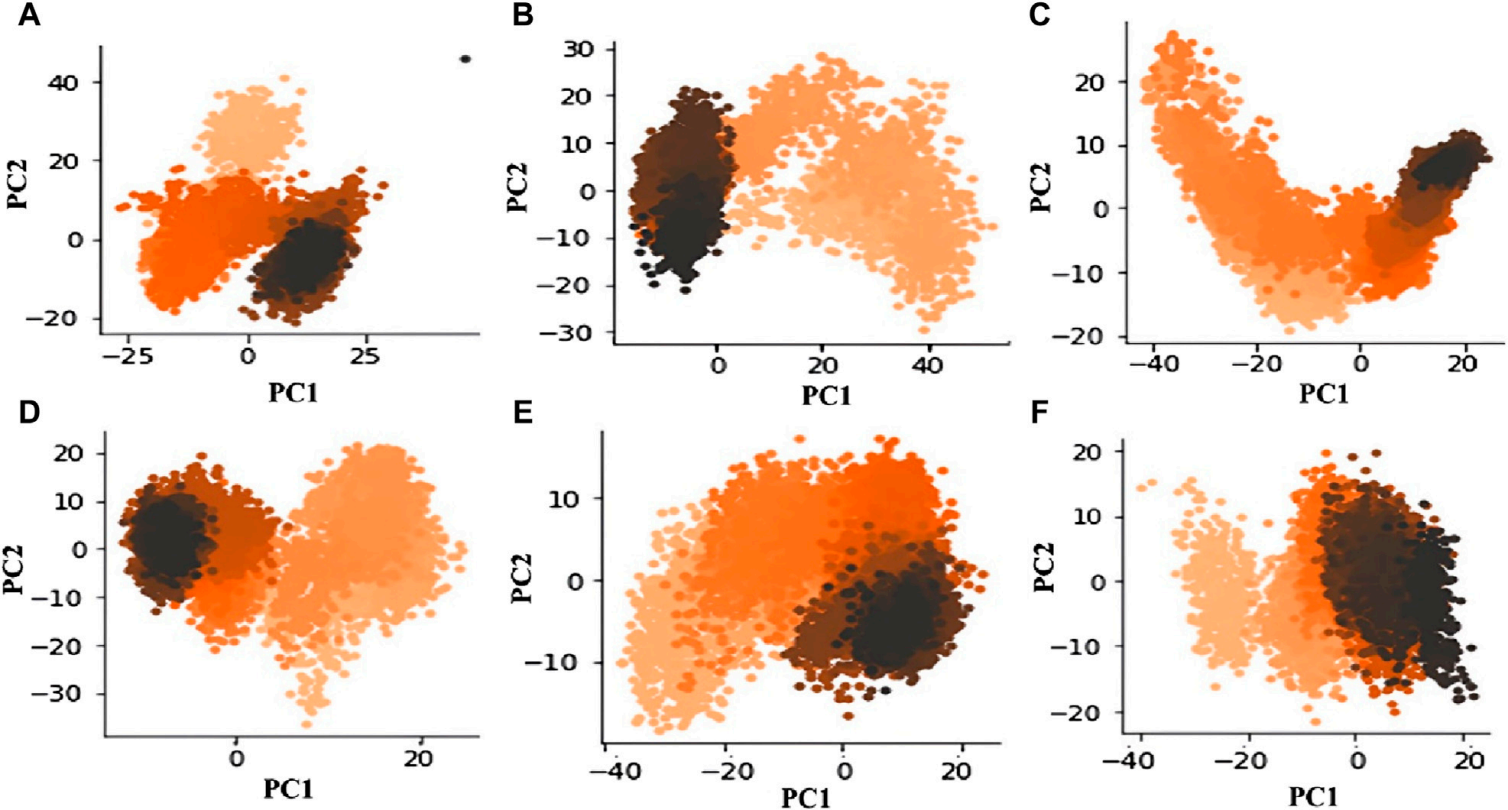
The conformational space is revealed by the scatter figure showing PC1 vs. PC2 for MIF bound to **(A)** ISO-1, **(B)** Ap1, **(C)** F9, **(D)** N16, **(E)** M12, and **(F)** Am22.

A statistical technique called PCA is used to distill data from large datasets. It is especially helpful for lowering the dimensionality of large datasets. PCA uses a mixture of orthogonal vectors to represent each frame’s coordinates, which simplifies trajectory data in MD simulations. The trajectory’s primary uncorrelated motion is captured by the first principal component, which has the biggest variance. Subsequent components account for increasingly lower variances (Pedregosa et al., 2011; Sittel et al., 2014). The analysis of the principal components essentially provides a distilled view of the critical dynamics, presenting frame-by-frame conformational fluctuations. Through the visualization of the PCA output, we can investigate relationships and trends between the structural characteristics that impact the complex’s stability, thereby revealing critical structural elements that contribute to the observed activity. Recently, our group applied PCA to explore complex conformational studies (Mushtaq et al., 2023). The outcomes of our analysis show that M12 and Am22 have relatively fewer conformational variations than the other entities. This is illustrated in the first principal component (PC1) to the second principal component (PC2) scatter plot (Figure 8). In particular, M12 and Am22 have a cumulative variance for the PC1 of 40% and 35%, respectively (Supplementary Figure S7). The plot indicates that the complex does not diffuse widely across conformational areas, which is noteworthy. On the other hand, the standard ISO-1 exhibited a distinct pattern. The scatter plot of principal components shows how ISO-1’s points are dispersed over the conformational landscape. Three different conformations were seen throughout the simulation. For ISO-1, the PC1 cumulative variance accounts for 45% of the variation. The dispersed arrangement of dots suggests a varied investigation of conformational regions, exposing different structural dynamics during the simulation. M12 and Am22 are less scattered along the PC1 axis, which depicts reduced structural dynamics in a conformational landscape.

### 3.5 Binding free energy calculation; determinant of ligand binding

Accurate free energy calculations are of central significance in molecular biology because free energies guide an array of cellular processes, from the signal transduction cascades to the biomolecular degradation. In most cases, these events involve ligand binding, thereby providing key insights into molecular recognition. In this relation, the estimation of free energies (ΔG_bind_) remains one of the key aspects while designing a ligand for a biological target in computer-assisted drug design (CADD). Herein, the ΔG_bind_ for the targeted flavonoids was calculated via a molecular mechanics/GBSA approach, offering a reliable and cost-effective alternative for such evaluations. The free energy calculations combined with the system’s energetics provided valuable insight into the determinants driving the complex formation. The extracted results for the ΔG_bind_ alongside the different energy components are listed in Table 1. As evident from the observed results, a more negative value of −21.63 kcal/mol was obtained for Am22, followed by F9 featuring a ΔG_bind_ of −13.09 kcal/mol, thus emphasizing a strong affinity towards the target protein. N16 displayed somewhat comparable results to the ISO-1, in parallel with the previous findings that highlighted its reduced stability compared to others. Further insight into the data indicated that the strength of the complex was mainly provided by the electrostatic contacts, which is also evident from the previous discussion highlighting the formation of multiple hydrogen bonds. The van der Waals interactions also served as critical determinants influencing ligand binding, which is attributed to the aromatic scaffolds present in the structures. In contrast, the positive values for ΔG_solv_ indicated their counteractivity in the formation of a tighter complex.

**TABLE 1:** The free energies (kcal/mol) of binding were calculated for the standard ISO-1 and selected flavonoids alongside the determinants driving the binding.

Energy components (kcal/mol)		Dietary flavonoids				
	ISO-1	Ap1	F9	N16	M12	Am22
van der Waals	−10.88	−19.64	−20.97	−17.76	−16.29	−31.62
Electrostatic	−29.76	−18.31	−22.82	−15.82	−25.50	−35.81
ΔE_surf_	−4.54	−4.43	−4.25	—	−4.45	—
ΔG_gas_	−40.64	−37.95	−43.79	−33.58	−41.79	−67.43
ΔG_solv_	33.55	24.87	30.70	25.95	34.80	45.80
Binding energy (∆G_bind_)	−7.10	−13.08	−13.09	−7.62	−6.99	−21.63

## 4 Conclusion

The pleiotropic nature of MIF and its potential role in the pathogenesis of several diseases, such as sepsis, cancer, and autoimmune-related ailments, is evident from the extensive literature. This emphasizes the attractiveness of MIF as a therapeutic target with pharmacological significance. Inspired by the anti-inflammatory and anticancer properties displayed by flavonoids, several studies investigated their ability to prevent MIF’s tautomerase activity. In this connection, potential dietary flavonoids with superior activity and low inhibition constants (IC_50_) have been reported by Yang et al. (2021). The precise mechanism for the observed impairment is still elusive. In this current work, a computational framework was utilized to explore the static and dynamic aspects of the binding mechanism of these dietary flavonoids. The target protein’s stability patterns, dynamic interactions, and time-dependent structural alterations were investigated in detail. The flavonoids featured good binding scores and interacted with Pro1A, Lys32A, Ile64A, Asn97B, and Asp95B residues from the tautomerase site. In general, these compounds exhibited stable binding during the molecular dynamic investigations. Nevertheless, N16 dislocated significantly from the tautomerase site. Although all the tested flavonoids demonstrated remarkable results, the results obtained for the potent inhibitors, particularly morin and amentoflavone, exhibited a good correlation with biological activity. The conformational landscape for these flavonoids revealed fewer structural variations. The obtained results are inconsistent with results derived from the other stability matrices utilized herein. The outcomes shed light on the functions and possible impacts of these flavonoids and MIF by offering a thorough mechanistic insight. These understandings could help direct future efforts focused on creating MIF inhibitors.

## Data Availability

The original contributions presented in the study are included in the article/Supplementary Material; further inquiries can be directed to the corresponding author.
