# Supplementary material for: Mechanistic insight into the mode of inhibition of dietary flavonoids; targeting macrophage migration inhibitory factor
Source: Front Mol Biosci. 2024 Jun 10;11:1414572. doi: 10.3389/fmolb.2024.1414572 (PMC11194440; doi:10.3389/fmolb.2024.1414572)
Supplement: Supplementary file 1 [file DataSheet1.docx]

**Mechanistic Insight into the Mode of Inhibition of Dietary Flavonoids; Targeting**

**Macrophage Migration Inhibitory Factor**

Ali Raza Siddiqui^1^, Mamona Mushtaq^2^, Madiha Sardar^1^, Lubna Atta^2^, Mohammad Nur-e-Alam^3^, Aftab Ahmad^4^, Zaheer Ul-Haq^2^*

^1^H.E.J Research Institute of Chemistry, International Center for Chemical and Biological Sciences, University of Karachi, Karachi 75270, Pakistan.

^2^ Dr. Panjwani Center for Molecular Medicine and Drug Research, International Center for Chemical and Biological Sciences, University of Karachi, Karachi-75270, Pakistan

^3^ Department of Pharmacognosy, College of Pharmacy, King Saud University, P.O. Box. 2457, Riyadh 11451, Kingdom of Saudi Arabia

^4^ Department of Biomedical and Pharmaceutical Sciences, Chapman University School of Pharmacy, Irvine, CA, 92618, USA

**Corresponding author:**

Zaheer Ul-Haq; zaheer.qasmi@iccs.edu

Office: +92-21 99261672


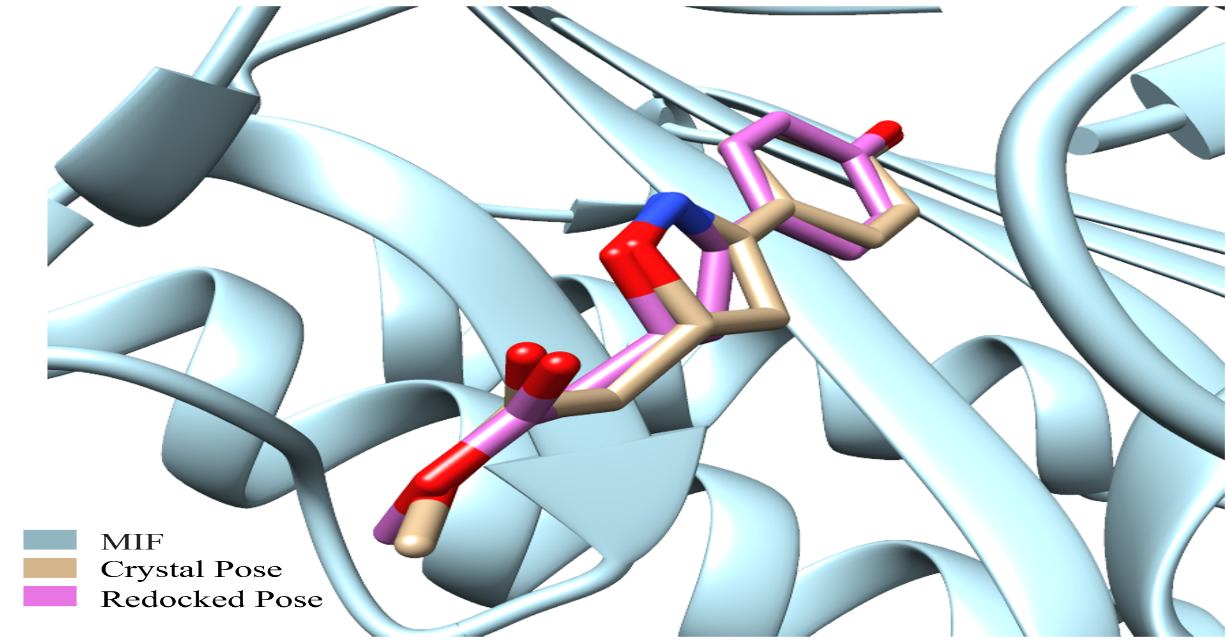


**Figure S1:** By superimposing the structural coordinates obtained from X-ray crystallography data with the resultant docked pose, the docking software and protocol were benchmarked. It was found that the resulting RMSD value was 0.50 Å.


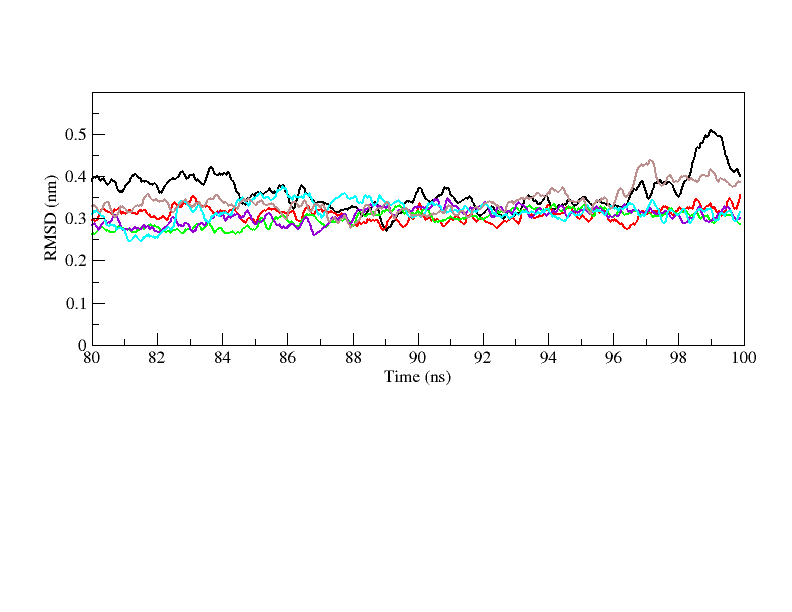


**Figure S2**: The Root Mean Square Deviation (RMSD) for the terminal stages of simulation indicating the overlapping pattern for Ap1 (Red), F9 (Green), N16 (Voilet), and M12 (Cyan). While the divergence exhibited by ISO-1 (Black) and Am22 (Brown).


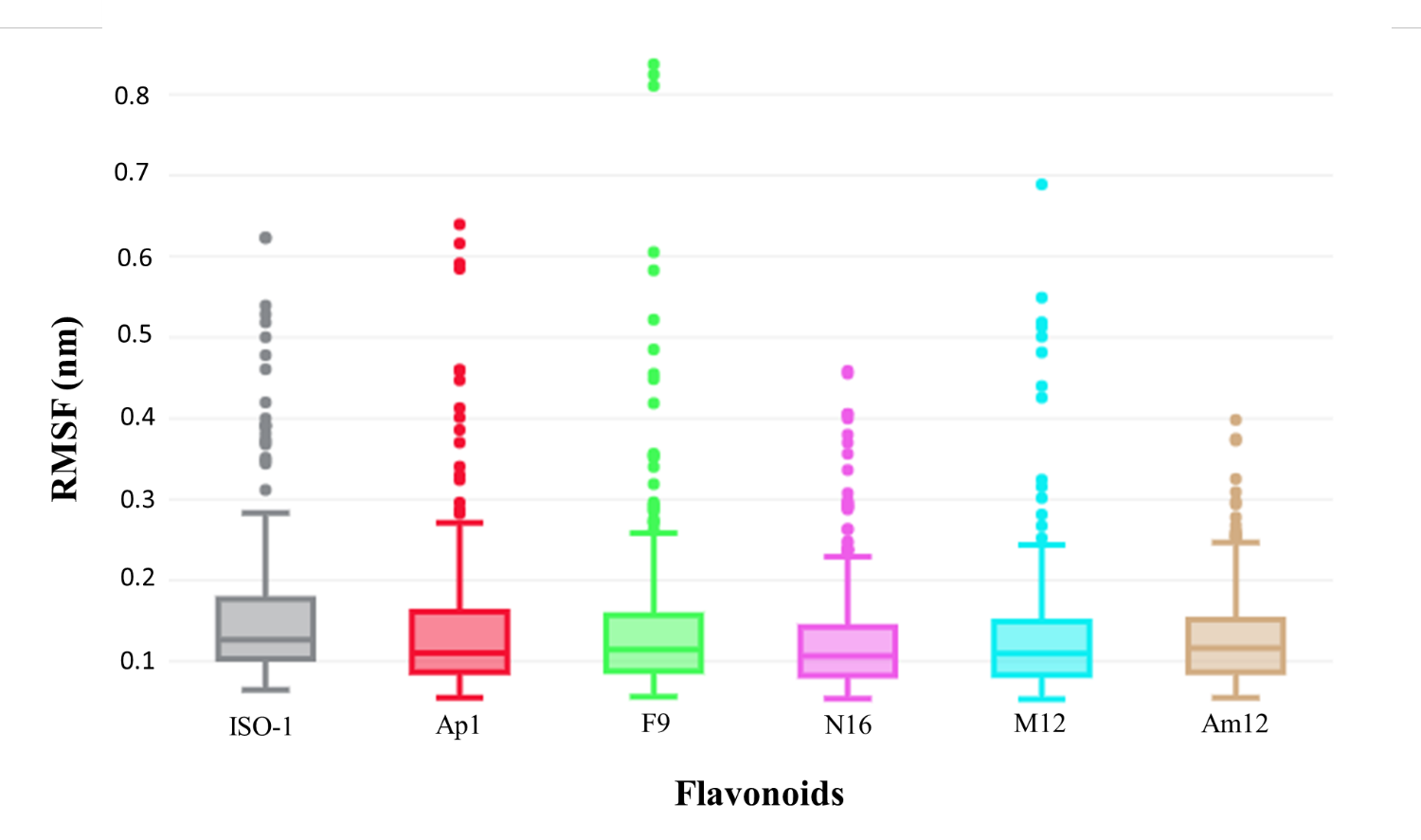

**Figure S3**: The boxplot showing the distribution of Root Mean Square Fluctuations (RMSF) experienced by the amino acid residues over time for the selected dietary flavonoids, indicating somewhat overlapping trends for the descriptive statistics.


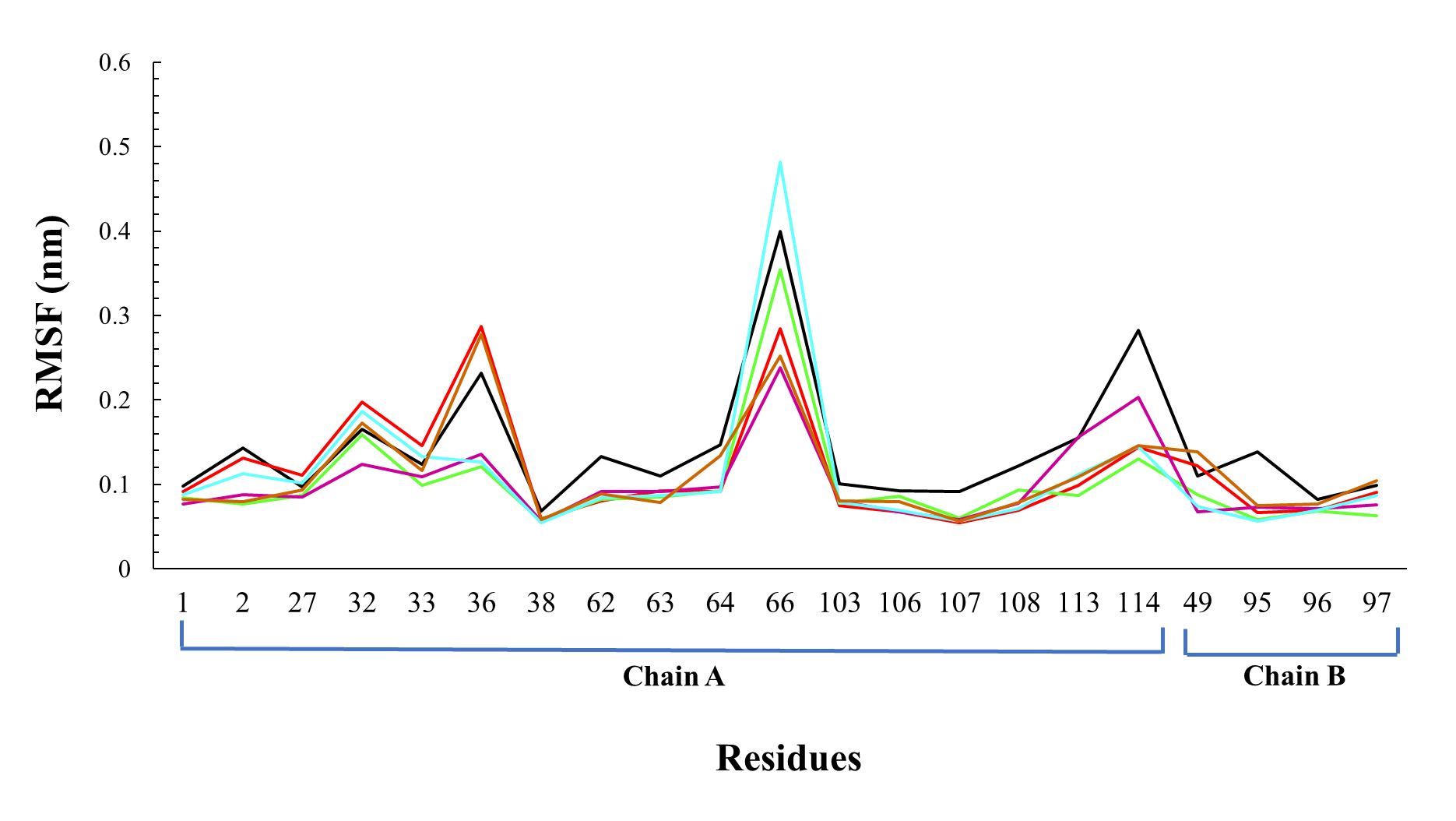


**Figure S4**: The Root Mean Square Fluctuations (RMSFs) for the residues lining the binding cavity for Ap1 (Red), F9 (Green), N16 (Voilet), and M12 (Cyan), Am22 (Brown), relative to ISO-1 (Black).


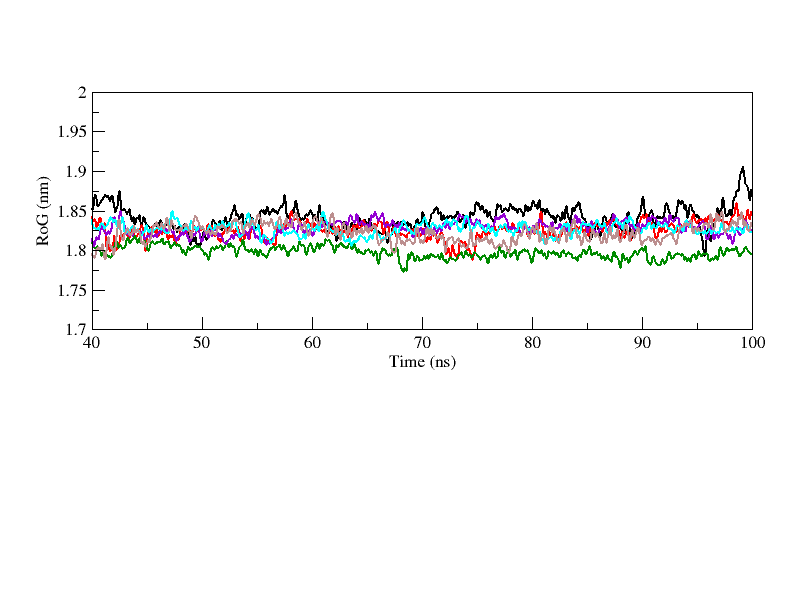


**Figure S5.** The RoG pattern during the converged state, for MIF bound to Ap1 (Red), F9 (Green), N16 (Voilet), and M12 (Cyan), Am22 (Brown) featuring an overlapping pattern for the flavonoids and stability compared to ISO-1 (black).


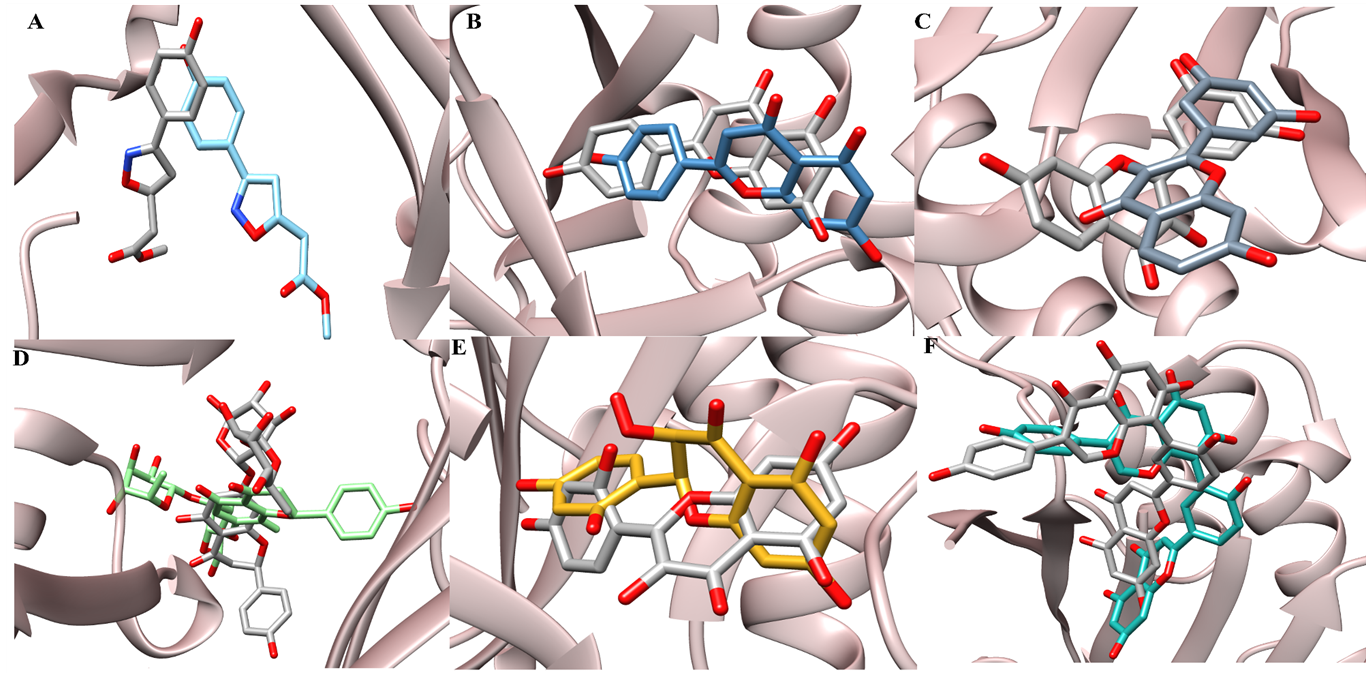

**Figure S‎6.** The superimposition of pre- and post-simulation binding poses attained by ligands in complex with MIF A) ISO-1, B) Ap1, C) F9, D) N16, E) M12, and F) Am22 flavonoids. The grey color stick model represents the docking poses for each of the ligand.

-


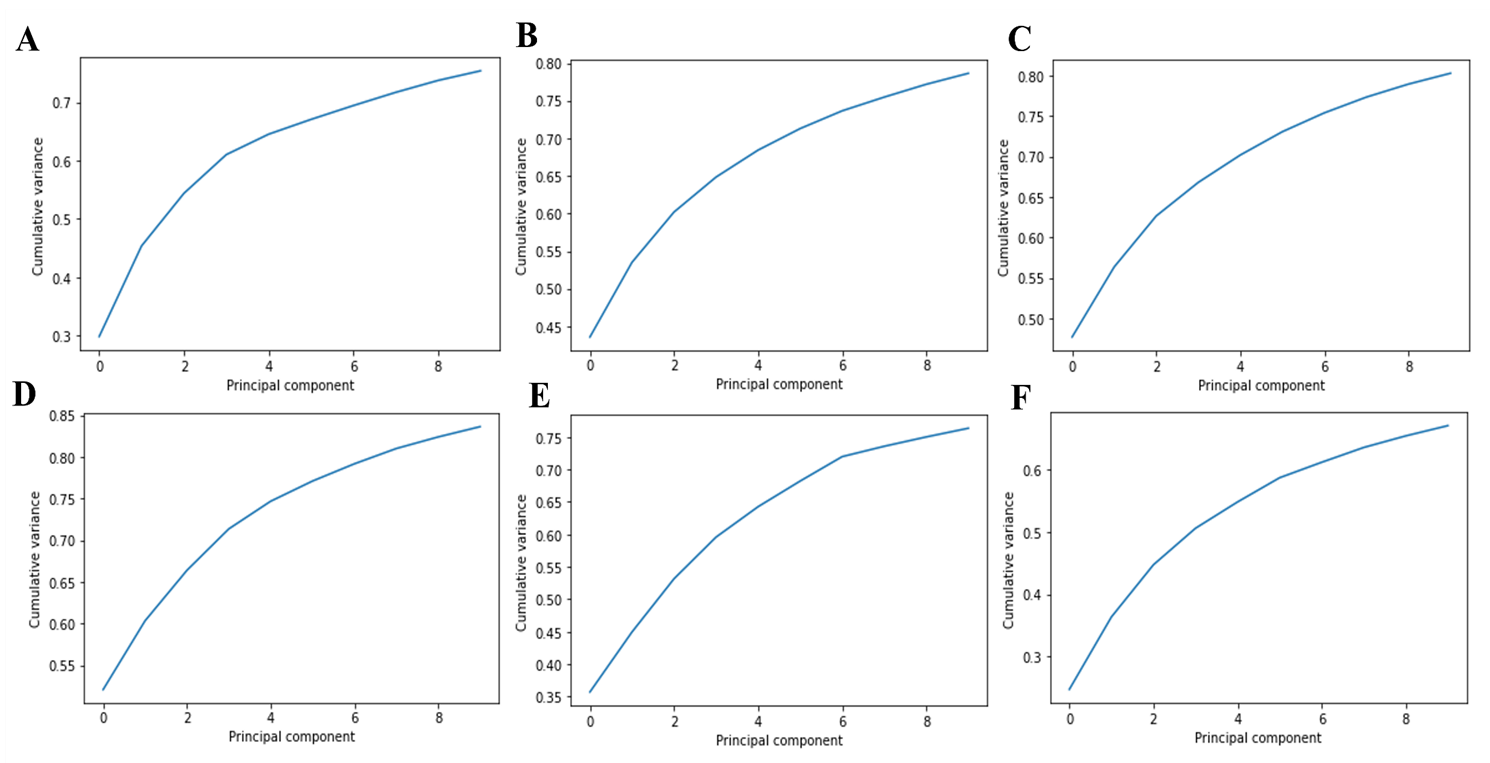


**Figure ‎S7**: The cumulative variance computed for the MIF bound to A) ISO-1, B) Ap1, C) F9, D) N16, E) M12, and F) Am22, offering insight into overall motion.

Table S1. An overview of the non-bonding interactions along with the binding pocket residues.

| Ligands | Pocket residue | | |
| --- | --- | --- | --- |
|  | Hydrogen Bonding | Hydrophobic interaction | Pi-stacking |
| ISO-1 | Pro1A,  Lys32A,  Ile64A,  Asn97B, Asn97B | Met2A,  Tyr36A,  His62A, Ile64A,  Phe113B | Tyr95B |
| Ap1 | Pro1A, Lys32A, Asn97B, Asn97B  . | Tyr36A, Ile64A,  Ile64A, Phe113A, Phe113A | Tyr36A,  Tyr95B |
| F9 | Pro1A, Lys32A, Ser63A, Ser63A, Ile64A, Asn97B | Tyr36A, Tyr36A, Tyr36A, Tyr36A, Ile64A, Phe113A, Phe113A | Tyr95B |
| N16 | Ala27A, Lys32A, Lys66A | Tyr95B | - |
| M12 | Pro1A | Tyr36A,  Tyr36A,  Tyr36A, Ile64A, Val106A, Phe113A,  Phe113A | - |
| Am22 | Lys32A, Tyr36A, Lys66A,  Asn97B,  Asn97B | Met2A, Lys32A, Tyr36A, Ile64A,  Ile64A,  Val106A, Phe113A,  Phe113A | Tyr95B |
